# An Information-Motivated Exploration Agent to Locate Stationary Persons with Wireless Transmitters in Unknown Environments

**DOI:** 10.3390/s21227695

**Published:** 2021-11-19

**Authors:** Daniel Barry, Andreas Willig, Graeme Woodward

**Affiliations:** Computer Science and Software Engineering, University of Canterbury, Christchurch 8020, New Zealand; andreas.willig@canterbury.ac.nz (A.W.); graeme.woodward@canterbury.ac.nz (G.W.)

**Keywords:** search and rescue, wireless transmitters, UAV, drone

## Abstract

Unmanned Aerial Vehicles (UAVs) show promise in a variety of applications and recently were explored in the area of Search and Rescue (SAR) for finding victims. In this paper we consider the problem of finding multiple unknown stationary transmitters in a discrete simulated unknown environment, where the goal is to locate all transmitters in as short a time as possible. Existing solutions in the UAV search space typically search for a single target, assume a simple environment, assume target properties are known or have other unrealistic assumptions. We simulate large, complex environments with limited a priori information about the environment and transmitter properties. We propose a Bayesian search algorithm, Information Exploration Behaviour (IEB), that maximizes predicted information gain at each search step, incorporating information from multiple sensors whilst making minimal assumptions about the scenario. This search method is inspired by the information theory concept of empowerment. Our algorithm shows significant speed-up compared to baseline algorithms, being orders of magnitude faster than a random agent and 10 times faster than a lawnmower strategy, even in complex scenarios. The IEB agent is able to make use of received transmitter signals from unknown sources and incorporate both an exploration and search strategy.

## 1. Introduction

In the last few years, unmanned Aerial Vehicles (UAVs) spurred substantial interest, as they can improve the delivery of existing services or enable provision of new services in a wide range of fields, including logistics [1], search and rescue (SAR) [2,3,4] public safety communications [5,6], infrastructure monitoring [7], precision agriculture [8,9], forestry [10,11], and telecommunications [12,13].

In this paper, we explore the use of UAVs in SAR scenarios in an unknown and possibly large terrain, with the intention of reducing time for locating victims. In particular, we consider a case where an individual UAV has to search for an unknown number of stationary persons in an outdoor area. We assume that the UAV is equipped with appropriate sensors to detect persons, e.g., based on a downward-facing camera using visible light or infrared. These sensors allow the UAV to decide the presence or absence of a person only in a relatively small area determined by the visual angle of the camera and the flying height of the UAV. With such a camera alone, to maximize the certainty that all persons will be located, the UAV would have to pick a path that is “dense”, i.e., which guarantees that each point is observed at least once through the camera (e.g., a ’lawnmower’ path). Such a dense path may require substantial time to travel. A key assumption in our work is that each person carries a wireless transmitter which emits signals frequently. The transmitter could, for example, belong to a cellphone, it could be a WiFi transmitter or an emergency beacon. We do not assume that the searching UAV has any a priori knowledge about the specific wireless technologies that any person may be using or channel properties, we only assume that the UAV is able to detect transmissions in a given frequency range, without having the ability to extract further information out of these transmissions. The searching UAV is equipped with appropriate receive circuitry, and hence it becomes possible for the UAV to detect the presence of a person from a distance, even when the terrain is cluttered or when the target (person) is outside the field of view of the UAV camera system. Conversely, when the UAV spends some time in a particular area and did not receive any wireless signals, then the UAV can conclude (with some uncertainty) that there is no person within the search scope or radio range of the UAV. Hence, the UAV does not need to spend any further time for a detailed inspection of the area using the downward-facing cameras, and it can move on to less well explored areas, with the intention of ultimately shortening the time required until the persons were located, therefore giving it a ’radio advantage’. While searching for persons, the UAV will also have to explore and map the terrain, which is not known in detail to the UAV when the search operation starts. We do assume, however, that the UAV knows some “bounding box” around the search area and can locate its position accurately via a technology such as GPS.

The overall goal in this application clearly is to minimize the average time until all the persons were located (minimum time search). At the same time, in a real application where lives may be at stake, certainty and thoroughness are clearly essential. This (and the case of persons not equipped with wireless transmitters) can be resolved by using a second UAV which just follows a “dense” path as described above (perhaps repeatedly), and which does not need to interact with the UAV searching for wireless transmitters, except perhaps to avoid collisions. Aspects related to this second UAV and the interaction between the two UAVs are not included in the scope of this paper.

The considered search problem is subject to substantial uncertainty. Besides the uncertainty about the terrain and the number of persons with wireless transmitters (which could be any natural number including zero), there is also uncertainty about a number of other factors, including the radio technologies used by the transmitters and their transmission powers (both of which could be different between different transmitters), and the uncertainty about the wireless propagation conditions. Transmit power and propagation conditions together determine the transmission range of a transmitter.

This paper makes the following main contributions:

- We present a discretized system model and formulation for the problem of searching an unknown number of wireless transmitters in a possibly large and unknown area with obstacles, including a radio model that includes limited-scale propagation characteristics like path loss. To the best of our knowledge, the search problem described in this paper was not widely considered in the literature.
- We describe the design of a novel search algorithm, which is loosely based on the information-theoretic concept of empowerment, and which incorporates limited assumptions for properties of the wireless channel. Our algorithm makes explicit the uncertainty present in the problem setting by modelling important properties of transceivers (e.g., their transmit power and frequency of signal/pulse emissions) and of wireless propagation as finite-range probability distributions, which may also encode any prior knowledge.
- We conduct a performance analysis of our algorithm and compare it against two baseline algorithms, one of which conducts a search along a “dense” path (note that we have not found any algorithm in the literature which addresses the same problem). In this analysis we assess the impact of important system parameters.

The remaining paper is structured as follows: we begin by introducing *related work* and discuss the related approaches that exist in the literature. We then give a description of the *system model*, which includes the search area, transmitter properties, search agent properties and the key performance measures. In the *algorithm* section we introduce our algorithm structure, the internal algorithm modelling, the model update function, the action selection process. Next, we introduce the two *baseline algorithms* we compare our search algorithm to, a random walk behaviour and a lawnmower behaviour. We then describe our *simulation setup*, specifically the parameters used for the experiments. After this, we provide our *results* for each batch of experiments, and more specifically, what was tested and what the results show. Lastly, we finish the paper with *discussion* and *conclusion* sections.

## 2. Related Work

The use of UAVs in real-world target location discovery showed promise in both single-agent and multiagent use cases [14]. Methods that focus on trajectory planning in a continuous space, where UAVs carry properties such as velocity, acceleration or other realistic movement modelling characteristics were shown to be effective in smaller search environments, but it remains unclear whether these search methods are able to scale to larger search environments. In the paper by Viseras et al. [15], we see that comparing a novel algorithm implementation to a random search implementation is an existing approach for benchmarking new algorithms, as we did in this paper.

One of the key assumptions made in this paper is that we are unable to locate the source of a detected transmitter, the agent only knows that it detected a signal and cannot uniquely identify it or the received power, an assumption made due to the inherent issues associated with determining distance. In work by Careem et al. [16], it is shown that it could be possible to approximate the location of a static target using an omnidirectional antenna by receiving a signal from a single device from multiple locations. To achieve this, they propose a method to uniquely identify the signal from the transmitter and make two or more detections, which are then used to determine an approximate location of the target.

For simple or known environments with sensor strength information, a particle filter method is quite effective in locating multiple targets with unknown properties [17]. With a priori information about the environment, a particle filter can be used in conjunction with an entropy reduction method to reduce the uncertainty of a single target’s location [18].

A similar problem is locating regions of interest (ROI), where the objective is to ensure some target exists within an area [19]. Given information about received sensor strength, we see some approaches map contours and perform a gradient descent method to locate the signal source. For this, one must assume a simple environment, accurate sensor strength measurements and assume there is no environment induced effect on the transmitter signal, such as occlusion. In practice, however, we expect signal loss and reflection, hence signal strength itself cannot be reliably used as a sole search method [20].

Whilst we mostly concentrate on minimizing time in our model, trajectory-based planning algorithms also can consider energy usage as an optimization parameter during search [21]. Given the current hardware limitations that limit flight time, energy use is an important consideration for search, although is outside of the scope of the work discussed in this paper.

Minimum time search (MTS) planners are algorithms that aim to minimize the time to achieve one or more goals [22]. Many such approaches adopt a Bayesian search method, where a common assumption is that the target is static, which of course is not true of all human search targets. The reason for this assumption is to reduce algorithm search complexity and is also the approach in this paper. Existing approaches assume a single target, some sensor that reveals information approximating to distance and a relatively small and simple search environment [23,24].

Discretizing the problem space allows for multiple classic search methods to be used. The environment is divided into patches (a series of smaller uniformly sized areas) and the UAV (agent) chooses from a finite set of actions in each discrete time step. There are several potential benefits of this approach, including the ability to simplify computation and take actions more quickly in the real world with limited onboard computation. Whilst this approach makes the exploration of larger, more complex spaces possible, it abstracts the problem space such that the energy consumption of the UAV cannot be easily calculated. An additional limitation is that for larger environments that were discretized, we see larger amounts of memory are used for storing information about the model.

One approach utilizes occupancy maps with a genetic algorithm for path planning [25]. In our opinion, whilst this approach is suitable for small search spaces, the computation time required to find suitable solutions in large search areas will become too high. It is not clear that this approach is able to larger environment sizes.

Another approach we considered is a partially observable Markov decision process (POMPD), where the agent looks to maximize a reward function given some action sequence in the next time steps [26,27]. Defining good reward functions is quite difficult and one typically has to make domain specific optimizations. It is difficult to implement a reward function that does not detect or locate a target directly given some action sequence, but instead choose less optimal near-future actions in favour of greater predicted gains. We suspect the algorithm we present in this paper could also be modelled as a form of POMPD hybrid with empowerment [28], where empowerment solves the reward function problem by means of intrinsic motivation (IM) [29]. The core idea of IM is that a reward function is not strictly specified and is simply the solving of an internal optimization problem, rather than an external one. As a result, richer and more complex behaviours can be achieved that are not task dependant.

In the approach described by Lanillos et al. [30], we see a more similar algorithm to the one we describe in this paper, where they describe updating a Bayesian model and the UAV agent maximizes the estimated probability of locating a target given an action sequence. This approach allows their UAV agent to effectively locate targets in a simple environment. Their algorithm does not address the issues of signal detection, obstacle modelling and manoeuvring or multiple target search problems. The algorithm is also unable to take paths that do not directly reward the agent within *n* time steps, if those paths allow the agent to discount large areas quickly, a problem addressed in our approach. Similarly to their approach, the authors of this paper also explored a *discounted time reward* in a previous work (to favour near-future discoveries), but found this negatively impacts the multitarget case [31].

## 3. System Model

In this section, we describe our main assumptions about the search area, wireless channel properties, and the search agent.

### 3.1. Search Area

We are given a rectangular search area, and we consider the search problem as a two-dimensional problem for the purpose of simplicity. The search area is discretized into quadratic *patches* of side length *l* meters, where *l* is typically thought of as 2 to 5 m (this is thought to be a reasonable assumption for an area that could be detected with a downward-facing sensor). The choice of *l* should be made such that radio signals have approximately constant strength over such a patch and the patch can be covered by the downward-facing camera (the height of the agent and the field of view (FOV) allow for appropriate coverage of the area).

We identify one corner of the search area as the “upper-left corner” and anchor a coordinate system in that corner, which counts in numbers of patches. In the horizontal or *x*-direction there are $L_{w}$ patches, and in the vertical or *y*-direction there are $L_{h}$ patches. We will express locations of agents or transmitters in terms of such patches, i.e., a location (x,y) refers to the *x*-th patch in horizontal direction and the *y*-th patch in vertical direction. Hence, *x* is an integer value between 0 and $L_{w}-1$, and *y* is an integer value between 0 and $L_{h}-1$. We refer to the overall search area or the set of all patches as

$$W=\left\{\left(x,y\right)\in N_{0}^{2}:0\leq x\leq L_{w}-1,0\leq y\leq L_{h}-1\right\}$$

There can be **obstacles** present in a patch and we assume that an obstacle occupies a patch completely or not at all. The agent cannot enter a place occupied by an obstacle, and a wireless transmitter also cannot be placed there. In this model, we assume that we do not have to deal with the case of a transmitter obstructed by something such as rubble. This is to reduce model complexity.

The numbers and positions of obstacled patches are not known to the agent a priori. To rule out pathological cases, we assume that transmitters are always reachable, i.e., they are not fully encircled by obstacles in a way that prevents an agent to enter the transmitter’s patch.

### 3.2. Transmitters and Wireless Propagation

We make several assumptions about transmitters in the world:

1. There can be zero, one, or more persons which may need to be rescued, each having a transmitter.
2. There is at most one transmitter and person pair in each patch.
3. Each of these persons is equipped with a wireless transmitter, which frequently emits wireless signals in one of a well-known set of radio frequencies. Each transmitter can be using one of a pre-defined set of wireless technologies (e.g., WiFi or Bluetooth or a cellular technology).
4. A particular wireless transmitter transmits its beacons with a transmit power *p* (in dBm) that is being taken from a finite set of allowable transmit powers $P\;=\;\left\{p_{1},p_{2},\ldots ,p_{N_{P}}\right\}$, with all the values given in dBm. The set P is known a priori to the agent.
5. A particular wireless transmitter transmits its beacons frequently, with an average beacon transmission rate of τ beacons per second, where τ is being taken from a finite set of allowable beacon transmission rates $T=\left\{\tau_{1},\ldots ,\tau_{N_{R}}\right\}$, with all values given in Hz. The set T is known a priori to the agent.
6. It is not known a priori to the agent how many persons or transmitters there are and what their chosen transmit powers and beacon generation rates are.

For each of the transmitters described above, we make the following assumptions about wireless signal propagation:

1. Suppose a transmitter is located in patch (x,y)—for the sake of definiteness let us say at the centre of the patch—and an agent is located in patch $\left(x^{\prime },y^{\prime }\right)$, again in the centre.
2. When there is an obstacle in the direct line-of-sight path between transmitter and agent, then the signal is completely blocked and the agent does not hear anything. This is a worst-case assumption.
3. Otherwise, the channel model between a transmitter in patch (x,y) and an agent in patch $\left(x^{\prime },y^{\prime }\right)$ follows a modification of the standard log-distance model [32], which accounts for the reference distance (which we assume here to be one meter). In this model, the total path loss at a distance d≥1m and for given path loss exponent γ and given path-loss *L* at the reference distance is given by (in dB):
  $$h\left(d|\gamma ,L\right)=\{\begin{array}{cc} L+10\cdot \gamma \cdot \log_{10}\left(d\right) & ,ifd\geq 1\\ L & ,\mathrm{otherwise} \end{array}$$
  We assume that neither the path loss exponent γ nor the initial path loss at the reference distance, *L*, are known a priori to the agent. However, as an approximation we assume that they both are taken from a finite set of eligible values. In particular, the path loss exponent γ is taken from the set $G=\left\{\gamma_{1},\ldots ,\gamma_{N_{E}}\right\}$, where $N_{E}$ is the number of allowed path loss exponents, and the initial path loss value *L* is taken from the set $L=\left\{L_{1},\ldots ,L_{N_{L}}\right\}$, where $N_{L}$ is the number of allowed path loss values. The sets G and L are known a priori to the agent. In this model, we did not include a shadowing term (often modelled as lognormal fading).
4. As a result, when the transmitter uses transmit power *p* (in dBm), and the distance between transmitter and agent is *d*, then the received signal power at the agent (in dBm) is given by
  $$P_{r}\left(d|p,\gamma ,L\right)=p-h\left(d|\gamma ,L\right)$$
  and the signal-to-noise-ratio (SNR) at the agent is then given by:
  $$S\left(d|p,\gamma ,L\right)=P_{r}\left(d|p,\gamma ,L\right)-N_{0}$$
  where $N_{0}$ is the total noise power (in dBm).
5. Finally, we assume that while the details of wireless transmission and propagation (transmit power *p*, beacon generation rate τ, path loss exponent γ, reference path loss *L*) are not known to the agent, there exists some threshold distance $R_{max}>0$ between transmitter and agent, beyond which the agent is guaranteed to not detect any transmission of a wireless signal. This value $R_{max}$ is known to the agent a-priori.
6. Notation: given a patch $\left(x^{\prime },y^{\prime }\right)$, denote by $N(x^{\prime },y^{\prime })$ the set of all patches $\left(x,y\right)\neq \left(x^{\prime },y^{\prime }\right)$ that have a Euclidean distance smaller than or equal to $R_{max}$ from $\left(x^{\prime },y^{\prime }\right)$, where the distance of two patches is meant to refer to the distance between their centre points.

### 3.3. Search Agent

We model a single search agent, which is initially placed in an unoccupied patch not containing an obstacle in the upper-left corner of the environment, from which every transmitter is reachable through a path of unoccupied patches. The search agent is a freely moving UAV in three dimensions (such as a quad-rotor drone, not a fixed-wing drone) with good manoeuvrability and the ability to perform sudden changes of direction. For simplicity, we ignore acceleration and deceleration characteristics.

The agent has a highly reliable and accurate source of location (such as that offered by a GPS receiver). Furthermore, we assume that the location tracking is good enough that the agent can at all times determine its location with high accuracy, specifically it’s location in the centre of a patch. We assume that the control mechanism required to achieve and maintain a given target location can be accomplished via a model-free adaptive control (MFAC) algorithm [33] or an active disturbance rejection control (ADRC) algorithm [34]. We consider problems regarding low-level motion planning and response to external disturbances outside the scope of this work and refer readers to the cited papers.

The agent is also expected to know the location of the reference “upper left” corner of the search area and the orientation of the reference frame in space. The upper-left corner is the location the agent starts its search from. The agent can always calculate which patch it is in from its current physical location in reference to this zero point.

The agent is equipped with three different kinds of sensors:

- A **downward sensor (D)**, like for example a camera, is mounted at the bottom of the agent and can inspect the current patch the agent is on. In particular, the downward sensor can determine with certainty whether there is a person/transmitter in the current patch or not.
- A **vicinity sensor (B)**, like for example a LIDAR, which allows the agent to determine its Moore neighbourhood, i.e., to determine reliably for all eight neighbouring patches (suitably modified for boundary patches) whether or not an obstacle is present in those. The vicinity sensor, however, does not give any information about the presence or absence of transmitters in the neighboured patches. The purpose of this sensor is to detect trees, buildings, power lines and large geographic features.
- A **radio sensor (R)** or **radio receiver** which includes an omnidirectional antenna. We assume that we did not receive demodulation circuitry for specific technologies (as these may add further weight to the agent, shortening its flight time), but rather that we can only detect the presence or absence of energy in certain predefined frequency bands. In other words, we can perform signal detection, but we assume that we do not attempt actual demodulation and extraction of data.

With this array of sensors, we use the radio sensor to discover whether we may be in the vicinity of transmitters, and the agent relies on the downward sensors to positively confirm the presence and location of a transmitter (In a related problem, one could leave away the downward sensors, but in this case one would have to rely on getting multiple signal detections at different locations (or using several parallel antennas) and on being able to obtain signal strength information—with this information, the agent could estimate the angle of arrival (AoA) [16] of the wireless signal and could aim to triangulate the transmitter position from repeated AoA estimations taken at different positions. Since the signal strength readings will be noisy, the AoA estimates will be noisy, too. Furthermore, since we only assume that we can detect the presence or absence of signals but cannot extract any information out of them, we would also have difficulties to distinguish whether multiple signal detections originate from the same transmitter or possibly from several transmitters located in the vicinity. This appears to become a very complex problem encapsulated by blind-source separation, and the downward sensor is one method of solving this problem).

We assume that the agent will not use receivers for specific technologies, but rather a technology-independent detector (e.g., based on measuring power in certain frequency bands). We do not make any specific assumptions about the detector, but we assume that there is a smooth and monotonically increasing function ϕ(·):R↦[0,1], which maps signal-to-noise-ratio (SNR) values given in dB to detection probabilities.

The agent moves with travelling speed *v* from the mid-point of one patch to the mid-point of a neighbouring patch. When the agent has reached the mid-point of the patch, it remains stationary for $T_{s}$ seconds to take sensor readings. At the end of this sensor reading time, the agent makes a decision as to which of the eight neighboured patches to visit next (with obvious adjustments for boundary patches). The time $T_{s}$ plus the time the agent requires to move completely through a patch at speed *v* gives the overall time $T=T_{s}+\frac{l}{v}$ that the agent stays in a patch. A tick is considered to be a single count of an agent “teleporting“ from the centre of one patch to the centre of another. We do not model the movement itself to allow the simulation to run faster than real time.

During the time period $T_{s}$ the agent:

- Uses its downward sensor to check whether a transmitter/person is present or not in patch $\left(x^{\prime },y^{\prime }\right)$.
- Uses the vicinity sensor to determine its Moore neighbourhood [35].
- Uses its omnidirectional antenna to detect wireless signals.
- Updates its internal model according to the update function, makes a decision about the next patch to go next according to the action function and then moves there.

We describe the sensor readings by a triple (D,B,R) where:

- $D\in \left\{0,1\right\}$ is the output of the downward sensor, where D=0 indicates that there is no transmitter in patch (x,y) and D=1 indicates that there is.
- *B* is the output of the vicinity sensor, it is an eight-tuple of Boolean flags indicating for each of the neighbouring eight fields whether or not an obstacle (or *block*) is present in that field.
- $R\in N_{0}$ is the output of the radio sensor, it gives the number of radio beacons that were detected during the time $T_{s}$ the agent listened for radio signals while being in patch $\left(x^{\prime },y^{\prime }\right)$.

### 3.4. Performance Measure

We measure the performance as the time taken to positively locate all transmitters (visit patches containing transmitters), but not necessarily having explored the entire environment. The agent is not aware of this measure, hence, they continue to explore the environment until otherwise halted.

## 4. Algorithm

In this section, we introduce our algorithm *Information Exploration Behaviour* (IEB). The algorithm is in part inspired by the concept of empowerment, which provides the equivalent of an objective function without needing to strictly specify one [29,36]. This means that an agent based on the principles of empowerment does not strictly need to be coded with the environment known a priori, making it suitable for complex and difficult-to-define environments. Works based on the concept of empowerment were successfully deployed to real-world agents that exhibit intelligent behaviour [37].

We begin with a brief overview of the algorithm in Section 4.1, define the agent’s memory structure in Section 4.2, give an overview of the update process in Section 4.3, define how an agent chooses an action in Section 4.4. Lastly, we discuss the algorithm complexity in Section 4.5.

### 4.1. Overall Structure

Our algorithm operates in a time-discretized perception-action loop. During the *t*-th time slot, the agent performs the following actions:

1. Move to the centre of a patch $\left(x^{\prime },y^{\prime }\right)$ and collect the sensor readings $S_{t}$ as described in Section 3.3.
2. Update an internal model representing the current belief about the presence or absence of transmitters and obstacles in all the patches the agent is able to observe at the position $\left(x^{\prime },y^{\prime }\right)$. More precisely, the model state from the previous round, $M_{t-1}$, is combined with the sensor readings of round *t*, $S_{t}$, to give an updated model $M_{t}=g\left(M_{t-1},S_{t}\right)$, where the function g(·) is the **model update function**.
3. After updating the internal model, an action $A_{t}$ is chosen out of the currently available actions in patch $\left(x^{\prime },y^{\prime }\right)$. The available actions are moving the agent into one of the neighboured, nonobstacled patches. More precisely, to calculate an action $A_{t}$ we apply an **action function** f(·) to the current model state $M_{t}$ and the current position: $A_{t}=f\left(M_{t},\left(x^{\prime },y^{\prime }\right)\right)$.

The algorithm has two distinct phases during search when computing f(·), the *Search Phase* and the *Discovery Phase*. As explained in Section 3.3 we do not assume that the agent is able to extract information out of received signals, and hence it cannot easily tell how many transmitters contribute to a sequence of received beacons or what their specific radio and propagation parameters may be. Hence, after hearing a signal, the agent can only say with confidence that there is at least one transmitter within a distance of $R_{max}$. The parameter $R_{max}$ should reflect the largest possible distance within which detection is feasible.

In the search phase, the agent takes actions that change its position in the environment and tries to detect new beacons, with its chosen path aiming to reduce overall uncertainty about transmitter locations as quickly as possible by generally preferring to go into less-well explored areas. Once a beacon is detected and its location confirmed using the downward sensor, we switch into the discovery phase, in which we perform a detailed and systematic search of the neighbourhood to locate the transmitter (or transmitters) of the beacon.

#### 4.1.1. Search Phase

The agent performs a search by maximizing predicted information gain based on the current model $M_{t}$ over a list of candidate paths, where a path is a list of eligible actions to be taken in the first, second, etc. step, and then selecting the first action of this sequence. The candidate paths are a random subset of all possible paths to reduce computational requirements. Selecting actions that maximize predicted information gain has the effect of minimizing model uncertainty, by confirming whether a transmitter exists in any given patch. In the case an action cannot be selected, we use a further-field search (see Section 4.4.4).

#### 4.1.2. Discovery Phase

In this case, we found the location of one or more transmitters in the local area, but cannot determine whether there are other transmitters in the vicinity. The radius $R_{max}$ surrounding the located transmitter may also contain one or more transmitters, meaning we must perform a more careful fine-grained search in the local area of the located transmitter to determine whether there is one or more transmitters. To achieve this, we do the following:

1. Clone our current model *M* into $M^{\prime }$—ignoring all patches that can be considered outside $R_{max}$ (these can be zeroed).
2. Set all values in $M^{\prime }$ that are not 0 or 1 (maximum certainty) to 0.5 (maximum uncertainty), indicating we unsure about the location of a transmitter in this location.
3. Use a minimal implementation of the IEB algorithm where only the predicted information within the area enclosed by $R_{max}$ is used to plan actions. During this search, the agent does not act on detected signals for model $M^{\prime }$. If signals are heard whilst performing the search, they are processed by the global model *M* and can be acted upon after the localized search, but are not acted on until the local discovery search is completed.
4. Search until all values in model $M^{\prime }$ are either 0 or 1, indicating each position was searched.
5. Update model *M* with the resulting internal belief values, found during the limited area search performed with $M^{\prime }$, such that the values within the area contained by $R_{max}$ are updated.

### 4.2. Model Structure

We start with defining the data structure of our model. As explained in Section 3.1, the environment is sub-divided into $L_{w}\times L_{h}$ patches, and we use the symbol M to refer to the set of all patches—we clearly have $\left|M\right|=L_{w}\cdot L_{h}$. For each patch (x,y)∈M we store in our internal model two pieces of information:

- A real number $M_{x,y}$ representing the current belief that a transmitter is in patch (x,y), hence $M_{x,y}\in \left[0,1\right]$. The value 0 represents the absolute certainty of there being no transmitter on this patch, and 1 represents absolute certainty of there being a transmitter on this patch (because it has been discovered through the downward sensor). For the case of zero probability, there can actually be two different reasons: the first reason is that we have detected an obstacle on that patch (recall that one of our assumptions is that transmitters and obstacles never share the same patch—see Section 3.1). The second reason is that there is no obstacle, but we have visited this patch in the past and have used our downward sensor to confirm that there is no transmitter. In doing this, we assume that our downward sensor is absolutely reliable, i.e., it makes no error in confirming the absence or presence of a transmitter.
- A Boolean flag $O_{x,y}$ which is only meaningful if $M_{x,y}=0$ and which indicates which of the previous two cases applies. Specifically, we set $O_{x,y}=1$ if there is an obstacle in patch (x,y), and $O_{x,y}=0$ if there is no obstacle. This information about the presence or absence of obstacles is mainly used in the generation of possible paths that the UAV can take.

At the start of the search, all the $M_{x,y}$ values are initialized with 0.5, reflecting maximum uncertainty as to whether or not a transmitter is present in any patch. Furthermore, all the $O_{x,y}$ values are initialized with 0. Note that there is no requirement that the $M_{x,y}$ values have to be normalized to sum up to one. This is helpful for a number of reasons:

- For large values of |M| a requirement for normalization would make most of the numbers $M_{x,y}$ very small, possibly leading to numerical difficulties.
- We do not have to carry out computations for the purpose of normalization after each update of the $M_{x,y}$ values.
- It is not obvious how normalization can be given a suitable interpretation if several transmitters are allowed.

We will often write the collection of the $M_{x,y}$ for all |M| patches as a matrix M or $M_{t}$, similarly we use the matrix O or $O_{t}$ for the collection of all flags $O_{x,y}$. *M* or $M_{t}$ denotes the state of agents internal model, which includes both M and O.

If we want to determine whether it is likely a yet to be located transmitter may still exist and whether the agent may stop searching, it is possible to evaluate the global uncertainty of the search area by calculating the likelihood of a transmitter remaining undetected. This would be achieved by checking the entropy of each patch containing a transmitter is significantly reduced, such that it can be said with high confidence that either a transmitter exists or does not exist for any given patch in the environment. It is also possible to keep searching until all patches within the environment were searched.

### 4.3. Model Update Function

We now describe how the model (all the $M_{x,y}$ and $O_{x,y}$ values in the table) are updated in response to a sensor reading (D,B,R) taken while the agent is in patch $\left(x^{\prime },y^{\prime }\right)$:

- Set $M_{x^{\prime },y^{\prime }}=D$, to record the absence or presence of a transmitter in the current patch. Furthermore, if D=0 (i.e., no transmitter) then also set $O_{x^{\prime },y^{\prime }}=1$.
- If one or more of the entries in the *B*-component of the sensor readings indicates an obstacle in the respective neighbour patch (x,y), set $M_{x,y}=0$, since there is an obstacle in that patch and no transmitter. Also, set $O_{x,y}=1$.
- If D=0, i.e., if no transmitter was detected in the current patch, we will have to update all patches $\left(x,y\right)\in N\left(x^{\prime },y^{\prime }\right)$ for which $M_{x,y}\notin \left\{0,1\right\}$ in a Bayesian way (see below, this part will have to account for the *R* component in the sensor readings) to change their current belief value $M_{x,y}$.
- If D=1, i.e., if we indeed have found a transmitter on the current patch, then all values $M_{x,y}$ for $\left(x,y\right)\in N\left(x^{\prime },y^{\prime }\right)$ are updated to be 0.5. This represents maximum uncertainty and incentivises the agent to search in this local area, allowing for the *discovery phase* described in Section 4.1.2.

In the remainder of this section we explain how we update the belief values $M_{x,y}$ for patches $\left(x,y\right)\in N\left(x^{\prime },y^{\prime }\right)$ for which $M_{x,y}\notin \left\{0,1\right\}$ in response to the observations made by the radio sensor.

We assume that the agent is in patch $\left(x^{\prime },y^{\prime }\right)$, and we consider a generic other patch $\left(x,y\right)\in N\left(x^{\prime },y^{\prime }\right)$. Our objective is to update the probability $M_{x,y}$ of finding a transmitter in patch (x,y) based on the radio observations made by the agent in patch $\left(x^{\prime },y^{\prime }\right)$. We denote by *d* the Euclidean distance between the mid-point of patch (x,y) and the mid-point of patch $\left(x^{\prime },y^{\prime }\right)$.

As discussed in Section 3.2, the transmitter transmits beacons at a given rate τ (measured in beacons per second). For our algorithm (but not our evaluation!) we now make the more specific assumption that the beacon transmissions have random, independent, and identically distributed intertransmission times, following an exponential distribution with rate τ. Hence, the beacon transmissions form a Poisson process [38] and the probability distribution for transmitting $B\in N_{0}$ beacons during time $T_{s}$ is given by

$$\mathrm{Pr}\left[B=b\right]=e^{-\tau T_{s}}\frac{{\left(\tau T_{s}\right)}^{b}}{b!}\;,\;b\in N_{0}$$

The agent does not know the beacon transmission rate τ adopted by the transmitter, but it knows a priori that the rate τ is chosen from the finite set T of allowed rates. In the absence of other information, the agent will assume that each of these rates is equiprobable.

The transmitter uses a fixed transmit power *p* (in dBm). Again, the agent does not know which transmit power the transmitter chooses, but it knows a priori that the transmit power is chosen from the finite set P of allowed transmit powers. In the absence of other information, the agent will assume that each of these transmit powers is equiprobable.

As discussed in Section 3.2, we are using a log-distance path loss model with path loss exponent γ and initial path loss *L*. Neither of these values are known to the agent, but the agent knows a priori that the path loss exponent γ is taken from the finite set G, and the initial path loss is taken from the set L. In the absence of other information, the agent assumes that each of the values in G and L are equiprobable.

We refer to one particular choice of τ∈T, p∈P, γ∈G and L∈L as a **configuration** and summarily write it as a tuple a=(p,τ,γ,L). We refer to C=P×T×G×L as the **configuration space**. Furthermore, let $T_{x,y}\left(a\right)$ denote the event that a transmitter of configuration a is located in patch (x,y). Assuming the particular configuration a=(p,τ,γ,L)∈C, the probability that the agent in patch $\left(x^{\prime },y^{\prime }\right)$ detects an individual beacon sent by a transmitter in patch (x,y) is given by

$$P_{a}\left(d\right)=1-\phi \left(S\left(d|p,\gamma ,L\right)\right)$$

where *d* is the Euclidean distance between patches $\left(x^{\prime },y^{\prime }\right)$ and (x,y).

Now, let $B^{\prime }$ be the random variable which counts how many of the *B* beacons transmitted by a transmitter in (x,y) during time $T_{s}$ are received by the agent when the configuration a=(p,τ,γ,L)∈C is being used. Since all beacon detection attempts are assumed to be independent of each other and all detections have the same detection probability, the Poisson process of rate τ underlying the generation of the *B* beacons is modulated by a binomial process, and the resulting process is again a Poisson process of rate

$\tau^{\prime }=\tau^{\prime }\left(d,a\right)=\tau \cdot P_{a}\left(d\right)$. Hence, the probability that the agent receives $B^{\prime }=b$ beacons when a transmitter of configuration a is located in patch (x,y) is given by

$$\mathrm{Pr}\left[\left.B^{\prime }=b\right|T_{x,y}\left(a\right)\right]=e^{-\tau^{\prime }\cdot T_{s}}\cdot \frac{{\left(\tau^{\prime }\cdot T_{s}\right)}^{b}}{b!}\;,\;b\in N_{0}$$

We form the **extended configuration space** $C^{\prime }=C\cup \left\{\partial \right\}$ where *∂* denotes the event that there is actually no transmitter in patch (x,y). We clearly have

$$\mathrm{Pr}\left[\left.B^{\prime }=b\right|T_{x,y}\left(\partial \right)\right]=\{\begin{array}{cc} 1 & ,\mathrm{if}\;b=0\\ 0 & ,\mathrm{otherwise} \end{array}$$

Now, suppose the agent observes $b\in N_{0}$ beacons while being in patch $\left(x^{\prime },y^{\prime }\right)$. Then, from Bayes’ law and with the law of total probability, the probability of there being a transmitter of configuration $a\in C^{\prime }$ in patch (x,y) can be updated as:

(1) $$\mathrm{Pr}\left[\left.T_{x,y}\left(a\right)\right|B^{\prime }=b\right]=\frac{\mathrm{Pr}\left[\left.B^{\prime }=b\right|T_{x,y}\left(a\right)\right]\cdot \mathrm{Pr}\left[T_{x,y}\left(a\right)\right]}{\mathrm{Pr}\left[B^{\prime }=b\right]}$$

$$=\frac{\mathrm{Pr}\left[\left.B^{\prime }=b\right|T_{x,y}\left(a\right)\right]\cdot \mathrm{Pr}\left[T_{x,y}\left(a\right)\right]}{\sum_{a^{\prime }\in C^{\prime }}\mathrm{Pr}\left[\left.B^{\prime }=b\right|T_{x,y}\left(a^{\prime }\right)\right]\cdot \mathrm{Pr}\left[T_{x,y}\left(a^{\prime }\right)\right]}$$

where $\mathrm{Pr}\left[T_{x,y}\left(a\right)\right]$ are the prior probabilities of there being a transmitter of configuration $a\in C^{\prime }$ in patch (x,y).

To update the belief $M_{x,y}$ that a transmitter is in patch (x,y) when the agent in patch $\left(x^{\prime },y^{\prime }\right)$ hears $b\in N_{0}$ beacons during the $T_{s}$ seconds listening time, we proceed as follows:

- We assume that the current value of $M_{x,y}$ represents our starting belief about there being a transmitter in patch (x,y). We furthermore will assume that each of the configurations a∈C (which notably does not include the *∂* configuration of there being no transmitter in this patch) is equally likely. With this in mind, we initialise our belief vector over the extended configuration space as follows:
  $$\mathrm{Pr}\left[T_{x,y}\left(a\right)\right]=\{\begin{array}{cc} 1-M_{x,y} & ,\mathrm{if}\;a=\partial \\ \frac{M_{x,y}}{\left|C\right|} & ,\mathrm{otherwise} \end{array}$$
- With this choice of the prior probabilities over $C^{\prime }$, we evaluate the Bayesian update Equation (1) for all $a\in C^{\prime }$. Denote the result for the specific configuration $a\in C^{\prime }$ by $U_{a}$.
- Update the overall probability of finding a transmitter in patch (x,y) to become
  $$M_{x,y}^{\prime }=\{\begin{array}{cc} 1-U_{\partial } & ,\mathrm{if}\;b=0\\ \min\left\{0.5,1-U_{\partial }\right\} & ,\mathrm{if}\;b>0 \end{array}$$
  When we actually do hear a beacon (i.e., b>0), then the case of there being no transmitter (configuration *∂*) is ruled out and $1-U_{\partial }$ becomes one, which is not meaningful in our setup, since the transmitter can also be in some other patch. Hence, in this case we limit the updated belief value to 0.5.

To conserve memory, we only use one variable $M_{x,y}\in \left[0,1\right]$ to represent our knowledge about patch (x,y), so we will not be able to distinguish between the (likelihoods of the) various configurations.

### 4.4. Action Function

#### 4.4.1. Generation of Candidate Paths

The purpose of generating candidate paths is that the agent can consider the result of *n* actions based on its current model $M_{t}$. We express a sequence of actions to be taken as $A=\{a_{t},a_{t+1},..,a_{t+n}\}$. Paths are of length *n* as this is the look-ahead parameter of the algorithm. We consider A to be a set of candidate paths. The agent considers all valid paths from its current position (x,y), where actions do not travel through known obstacles or boundaries (as defined by the agents internal model $M_{t}$). The algorithm evaluates the benefit of each path by calculating the expected information gain from each path using IEB.

To reduce the inherent computational complexity for large search depth values *n*, we do not search all possible action sequences. Methods such as upper confidence trees (UCT) Monte Carlo tree search exist for better than random tree search reduction, but we select a random search to reduce the complexity of this work [39]. For a given value of *n* we define a maximum number of action sequences to search, $A_{max}$, and a count of generated action paths $A_{count}$. We then probe our action space with the following steps:

1. Initialise $A_{best}$ to be some random action sequence that starts with an action $a_{t}$ with a visit-able patch. $A_{count}$ should be zero as paths are yet to be generated.
2. For each available action $a_{t}$:
  – Generate a random candidate path *A*.
  – We ensure the path is valid (inside environment bounds and does not go through the known location of an obstacle) by stepping through each action starting at $a_{t}$ and ensuring it is a possible legal state. Actions which cause an invalid state are randomized until no path conflicts are detected.
  – Evaluate the effectiveness of the path (see Section 4.4.2). If the expected information gain is better than that of the current best path, set $A_{best}=A$.
3. If $A_{count}=A_{max}$, then end the search and select $A_{best}$, with the agents next action $a_{0}$. If $A_{count}\leq A_{max}$, repeat from the previous step.

The cost of generating random paths in each loop can be greatly reduced by pregenerating paths and testing their suitability given the current state of the agent. The suitability constraints include generating paths that do not occupy the patches of known obstacle positions and do not exceed the boundary of the environment. Once we deplete the pregenerated paths we use the slower random path generation. Dependant on the *n*-step value and number of random paths, it is likely that most paths will be unique. In the case where they are not unique, this does not affect the candidate path selection.

#### 4.4.2. Calculating IEB of a Single Candidate Path

Suppose our agent is on patch (x,y) and we are processing the effectiveness of a single action sequence $A=\{a_{t},a_{t+1},..,a_{t+n}\}$. Firstly we operate on a clone of our current model $M_{t}$ which we represent as $M_{t}^{\prime }$. A high level view of the algorithm is as follows:

1. We use a quantity related to the total uncertainty in $M_{t}$ to provide a comparison. The quantity introduced below is a variant of the well-known information-theoretic notion of entropy [40], modified to account for working with a belief vector instead of a probability distribution.
2. Apply the action sequence *A* on the cloned model $M_{t}^{\prime }$, producing $M_{t+n}^{\prime }$, where each location the agent visits it is assumed that maximum information gain is achieved (each visited location in $M_{t+n}^{\prime }$ set to zero). This model represents the expected result of having performed the actions.
3. Evaluate the entropy of $M_{t+n}^{\prime }$.
4. Calculate the expected information gain *I* based on the action sequence *A*.

As an agent, we simply want to maximize our expected information gain, which is the path *A* with the largest information gain *I*. Once this is chosen, the agent simply performs the first action in the sequence, $a_{t}$. The process in more detail is as follows:

1. Calculate the entropy of our current internal model (The entropy calculation H() is not strictly entropy as we operate on a belief vector and not probabilities. We adopt the same notation as the calculation and purpose is otherwise same. In the remainder of the paper, we will simply refer to *entropy* and *model entropy*):
  $$H\left(M\right)=-\sum_{(x,y)\in M}M_{x,y}\log M_{x,y}$$
  where we assume xlogx=0.
2. As the agent visits a given location, it can be assumed that all information was observed in that patch. Therefore, we zero the positions the agent visits using the action sequence *A* (by setting their $M_{x,y}$ values to zero—this amounts to assuming that we do not find a transmitter in these patches) and store the result in $M^{\prime }$.
3. Calculate the entropy of the predicted internal model:
  $$H\left(M^{\prime }\right)=H\left(M|A\right)=-\sum_{(x,y)\in M}M_{x,y}^{\prime }\log M_{x,y}^{\prime }$$
4. The expected information gain is the difference in entropy between the calculated current information and expected information:
  $$I\left(M;M^{\prime }\right)=H\left(M\right)-H\left(M|A\right)$$

#### 4.4.3. Evaluating IEB for Action Sequences

The question our agent wants to answer at each time step is “which action should I take next?”. We answer this by comparing the action sequences from the previous step. We denote E as the IEB value and calculate each action as the following (which shares similarities with *n*-step empowerment [28]):

$$E=C\left(p\left(M_{t+n}|a_{t}^{n}\right)\right)=\max_{p(a_{t}^{n})}I\left(A_{t}^{n};M_{t+n}\right)$$

Essentially, we pick the path that yields the greatest expected information gain and choose to perform the first action of the sequence $a_{t}$.

#### 4.4.4. Horizon Problem

There exists a corner case when the algorithm is evaluating candidate paths, where there are no paths within the current *n*-step horizon that give any significant information—for example because the agent is located in an area that has already been searched exhaustively. This presents itself as the maximum channel capacity for the best action $a_{t}$ being equal to one or more other actions. The predicted information gain for a given action may be zero, indicating that there is no expected information gain in the local vicinity, or equal value, where there is no preferred information. In this case, it is not clear which action is to be selected next as there is no clear preference.

We refer to this condition as the agent being entropy-deprived. In this case the agent needs to travel a longer distance into areas where there is still potential information gain. A trivial approach would be to switch the agent into a pure random walk mode until it finds itself in a more information-rich area, but we expected this to be suboptimal and opted for another approach.

Our approach solves two problems: identifying an “interesting” target area, and moving the agent there. For the latter problem we use a standard algorithm from robotics, the wave-front path planner described in [41] and do not comment on it any further here due to space reasons. Instead of simply identifying the patch (x,y) with the highest entropy (uncertainty) value $H(M_{x,y})$ and go there, we prefer to visit larger unexplored areas with substantial total entropy. We aim to achieve this with the following approach:

- Our model *M* stores belief values. For each such belief value $M_{x,y}$ we determine its entropy value $H(M_{x,y})$ and use these entropy values as input for calculating a standard data structure in computer vision, the so-called summed area table [42]. We do not discuss the details of this data structure here due to lack of space, but its main use for our paper is to calculate for an arbitrary given rectangular area the sum of the entropies of all patches contained within that area.
- We use the ability to query the summed entropy of rectangular areas in the following iterative algorithm (quad-tree search): We start by subdividing the overall rectangular environment into four (first-order) quadrants. For each first-order quadrant we query the summed-area table to identify the first-order quadrant with the largest summed entropy. The resulting first-order quadrant is then again sub-divide into four (second-order) quadrants. We again identify the second-order quadrant with the largest summed entropy, subdivided this further, and so on. We stop our algorithm when the quadrant size is reduced to a single patch and return the quadrant coordinates with the largest entropy.

Once a target patch was identified, we use the wave-front planner algorithm to determine the next steps going there. This algorithm is capable of planning paths around known obstacles. We do not insist for the agent to actually reach the target patch, but we switch back to normal IEB behaviour once the agent enters an area where the condition of the agent being entropy-deprived ceases to apply.

### 4.5. Computational Complexity

The complexity of the algorithm largely depends on the scenario in which it is operating in and the algorithm’s mode of operation. We will therefore consider several scenarios and approximate an upper bound of complexity.

- *IEB normal operation*—in this case, we are applying our standard algorithm (in both the *search* and *discovery* phases). The process is as follows:
  - *Model update*—we update the agent’s internal belief values. Whether or not a signal is heard, we must update all internal values within $R_{max}$ to reflect this, so the update cost is $O(R_{max}^{2})$.
  - *Calculate next action*—calculate for a fixed number of *n*-step paths the potential entropy gain. This computation is made more complicated when a candidate path turns out not to be feasible (note that each path will be feasibility-checked, which is O(n)). For *k* paths, we compute O(k∗n) actions. In this case, we use a randomised backtracking method to find a feasible path, and as a result of backtracking we may have to inspect approximately 4n patches to form a path in the worst case.
- *Deprived entropy case*—in this case, our *n*-step lookahead algorithm is unable to determine near-future information gains, and no immediate action is favourable. This can occur for example if all local patches are explored and the agent can say with certainty that there is no local information to be gathered. In this case, we perform our *horizon search* algorithm. The process is as follows:
  - *Quad-tree calculation*—we have to calculate this summed-area table and to perform the “quadtree search”. This is $O(n^{2})$, as the entire agent’s belief vector needs to be considered. Once this is calculated, the result can be cached and reused.
  - *Find peak entropy area*—here, we calculate the location of a single patch, which is located within the largest area of entropy. Because of the previous calculation, this search is $O(\sqrt{n})$.

Due to the nature of the search modes, which are also affected by the environment, we do not specify an overall algorithm complexity. For example, *deprived entropy case*, a rare but possible scenario, will clearly require much more computation to derive a next-action $a_{t}$.

## 5. Baseline Algorithms

To test the effectiveness of the algorithm, we compare against two different agents:

- **Random walk**—at each time step the agent is given a set of possible actions *A*, where it randomly selects an action with a uniform distribution to be taken from a set of possible actions. This agent also works in obstructed environments. We consider an upper bound for the time taken for the random walk to cover all patches is equivalent to the so-called *covering time*, which for a two-dimensional n×n torus is asymptotically $∼O(n^{2}{\left(\log n\right)}^{2})$ [43] (The *covering time* is a more appropriate measure for searching the entirety of a torus-shaped environment. The complexity described should be treated with care and used only to give some approximate order of magnitude). This is of course a simplification of our problem space, as the action space *A* can be reduced by invalid actions generated by the sides of the designated environment or obstacles.
- **Lawn mower**—the agent performs a “lawn mower”-like action, where it plans a path that goes through each patch in the open field, without considering the radio information. This agent is unable to navigate around obstacles and is therefore restricted to the open field scenario. The upper bound for an open field is therefore $O(n^{2})$.

For each agent, a suitable number of maximum iterations were set that none of the agents reached this number of ticks.

## 6. Simulation Setup

The simulation environment was designed to avoid situations where transmitters are impossible to reach by borrowing from maze design, particularly by using a randomized depth-first search recursive-stack backtracker [44]. A maze is created such that obstacles occupy an entire patch, which is followed by randomly removing obstacles from patches until the desired ratio of obstacles in the environment is obtained. This is not an efficient environment setup, but is simple and only needs to be run once per simulation. The maximum possible ratio of obstacles to nonobstacles is ≈$\frac{1}{2}$ in a full environment. Greater numbers of obstacles resembles the internals of a building, whereas a lower number of obstacles is more representative of an open landscape.

The agent is spawned in the upper-left of the environment in patch (0,0), which is guaranteed to be available as the obstacle placement algorithm starts there also. The transmitters are then randomly placed in the environment in places where there is not an obstacle or another transmitter, until the desired number of transmitters is reached. The transmitter properties are assigned randomly with equal probability for any configuration. Each transmitter in the environment is unique in placement position, but is not guaranteed to be unique in transmitter properties.

In addition to the values discussed in Table 1, we define several constants. The environment size is $L_{w}=500$ and $L_{h}=500$, for a total of 250,000 patches. We set the total noise power to $N_{0}=-120\;dBm$ and have the agent assume the worst $R_{max}$ from the configuration space. The configuration space C is varied for each experiment.

The algorithm’s *n*-step is 10, with the number of randomly generated paths set to 5000. Simulations are allowed to run for a maximum of 500,000 ticks (none have come close to reaching this). The patch side length is set to l=2 m. We run 1,000 replications per data point and we report the averages, with standard deviation indicated.

In each experiment the transmitters are randomly placed and the agent is unaware of the transmitter properties. If applicable, the obstacles are also randomly placed such that

## 7. Results

We conduct three different types of experiments, the *Baseline* (Section 7.1), *Varying Transmitters* (Section 7.2), *Varying Obstacles* (Section 7.3). The *Baseline* experiments allow us to compare the two baseline algorithms described in Section 5 with the IEB agent. As both baseline algorithms are unable to be tested in every experiment, we utilize this scenario to give a fair comparison. In the *Varying Transmitters* scenario, we observe the IEB agent’s response to unknown varying transmitter configurations. This allows us to inspect how each transmitter configuration affects the agent’s ability to locate all transmitters in the environment. For the *Varying Obstacles* scenario we compare the IEB agent to the random agent in order the gauge the agent’s ability to locate transmitters within the environment with the addition of increasing numbers of obstacles. For each result we calculate the standard deviation.

### 7.1. Baseline

The focus of the first experiment is to consider an “ideal” scenario, where we consider a simple configuration space, no obstacles and compare to our baseline algorithm, the lawnmower behaviour. Here, we consider only one type of transmitter whose properties are fixed, but unknown to the agent.

**Figure 1:**
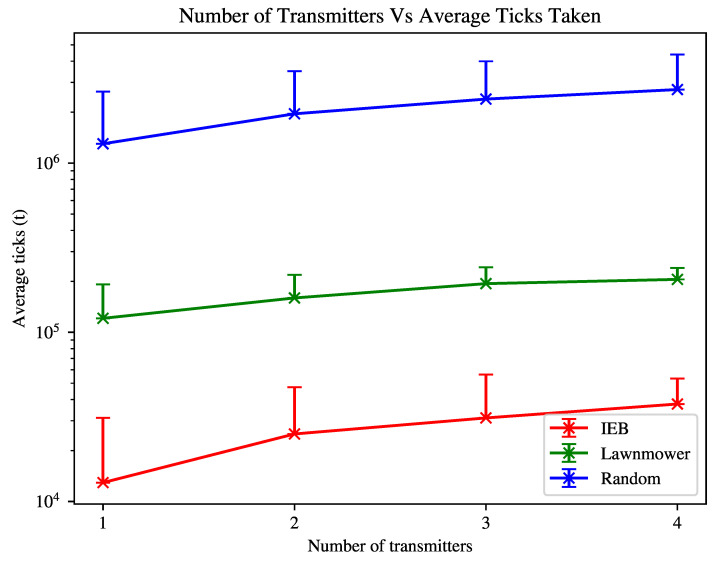
Time taken to find all transmitters within environment. Error bar indicates $\sigma^{2}$ standard deviation.

In the first experiment shown in Figure 1, we have the following environment variables: transmitters = 1,2,3,4, obstacles = 0. The agent’s n-step is 10. The configuration space, C, is initialised as follows: L = $\left\{50\;\mathrm{dB}\right\}$, G = $\left\{2\right\}$, P = $\left\{100\;\mathrm{mW}\right\}$, T = $\left\{10\;\mathrm{Hz}\right\}$.

In Figure 1 we see that the IEB agent is able to find the transmitters in the environment approximately one order of magnitude faster than the lawnmower agent, and approximately two orders of magnitude times faster than the random agent. For both algorithms the average search time increases with the number of transmitters as a larger amount of the environment is required to be explored on average to locate all transmitters.

### 7.2. Varying Transmitters

Next, we consider expanding the configuration space for the second experiment in an open-field (no obstacles), to test the agent’s ability to detect transmitters when no prior transmitter configuration is assumed. We then compare the agent’s ability to detect and locate transmitters of a given type. This gives us some indication as to the “radio advantage” offered to the IEB agent over the lawnmower agent, given the ability to detect beacons. We also observe the difference that each particular transmitter property has on the agent’s ability to locate transmitters effectively.

**Figure 2:**
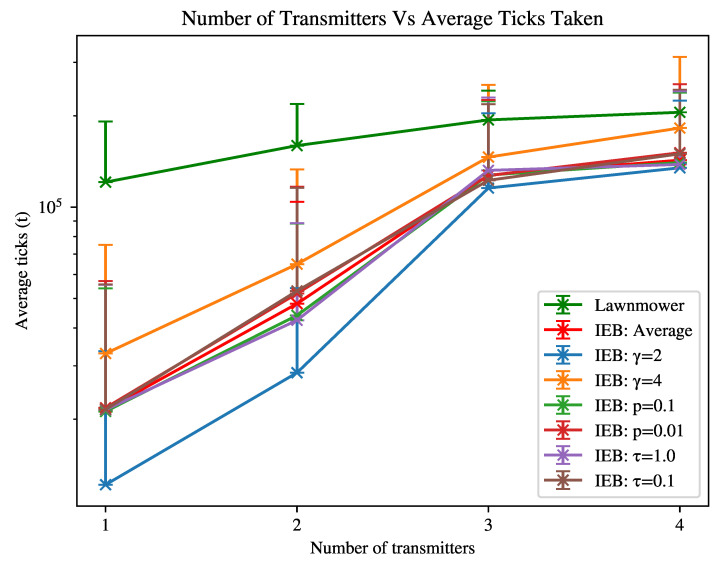
Time taken to find all transmitters within environment with varied transmitter properties. Error bar indicates $\sigma^{2}$ standard deviation.

In the second experiment as shown in Figure 2, we have the following environment variables: transmitters = 1,2,3,4, obstacles = 0%. The agent’s n-step is 10. We collect results for the following combinations of parameters: C is L = $\left\{50\;\mathrm{dB}\right\}$, G = $\left\{2,4\right\}$, P = $\left\{10\;\mathrm{mW},100\;\mathrm{mW}\right\}$, T = $\left\{1\;\mathrm{Hz},0.1\;\mathrm{Hz}\right\}$. These values were selected by experimentation and offer a somewhat realistic set of transmitter properties, allowing us to assess the relative impact of each parameter.

The configurations are as follows (where over-bars represent averages over the given set):

- *Average*, where L = 50dB, G = $\bar{\left\{2,4\right\}}$, P = $\bar{\left\{10\;\mathrm{mW},100\;\mathrm{mW}\right\}}$, T = $\bar{\left\{1\;\mathrm{Hz},0.1\;\mathrm{Hz}\right\}}$.
- γ=2, where L = 50dB, G = 2, P = $\bar{\left\{10\;\mathrm{mW},100\;\mathrm{mW}\right\}}$, T = $\bar{\left\{1\;\mathrm{Hz},0.1\;\mathrm{Hz}\right\}}$.
- γ=4, where L = 50dB, G = 4, P = $\bar{\left\{10\;\mathrm{mW},100\;\mathrm{mW}\right\}}$, T = $\bar{\left\{1\;\mathrm{Hz},0.1\;\mathrm{Hz}\right\}}$.
- p=0.1, where L = 50dB, G = $\bar{\left\{2,4\right\}}$, P = 100mW, T = $\bar{\left\{1\;\mathrm{Hz},0.1\;\mathrm{Hz}\right\}}$.
- p=0.01, where L = 50dB, G = $\bar{\left\{2,4\right\}}$, P = 10mW, T = $\bar{\left\{1\;\mathrm{Hz},0.1\;\mathrm{Hz}\right\}}$.
- τ=1.0, where L = 50dB, G = $\bar{\left\{2,4\right\}}$, P = $\bar{\left\{10\;\mathrm{mW},100\;\mathrm{mW}\right\}}$, T = 1Hz.
- τ=0.1, where L = 50dB, G = $\bar{\left\{2,4\right\}}$, P = $\bar{\left\{10\;\mathrm{mW},100\;\mathrm{mW}\right\}}$, T = 0.1Hz.

Figure 2 shows that the IEB agent is slower to find all transmitters in the environment when the configuration is less favourable (beacon is received less reliably), where the “radio advantage” (the advantage the IEB agent has from detecting transmitters) is decreased when the number of transmitters is increased. In the least favourable case scenario, the IEB algorithm performs approximately as well as the lawnmower agent. The most influential parameter for search times is the path loss parameter γ∈G, affirming that the agents ability to detect a transmitter is important to decreasing the time to locate each transmitter in the environment.

### 7.3. Varying Obstacles

For our third experiment, we look to observe the effect of obstacles on the agent’s search behaviour. In this scenario we consider only one transmitter is to be found, and instead add obstacles to the environment. In this scenario, we are unable to compare the agent against the lawnmower agent, as it is unable to navigate around obstacles.

**Figure 3:**
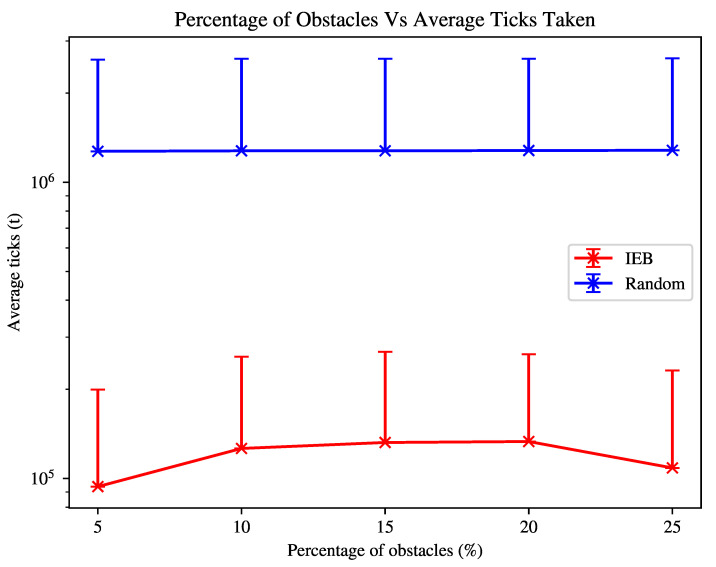
Time taken to find a single transmitter within an environment with an increasing number of randomly placed obstacles, where average time is shown. Error bar indicates $\sigma^{2}$ standard deviation.

For the experiment shown in Figure 3, the environment is initialised such that transmitters = 1, obstacles = 5%,10%,15%,20%,25%. The agent again has an n-step = 10. C is defined as: L = $\left\{50\;\mathrm{dB}\right\}$, G = $\left\{2\right\}$, P = $\left\{100\;\mathrm{mW}\right\}$, T = $\left\{10\;\mathrm{Hz}\right\}$. The agent again assumes the least favourable configuration of transmitter properties from the configuration space.

Figure 3 shows that increasing the number of obstacles in the environment increases the time taken to find transmitters, as the agent needs to also navigate around obstacles and the obstacles ’block’ the transmitter signal, significantly decreasing the agent’s ability to detect a transmitter and adjust its search strategy based on this information. As also shown in Figure 2, the agent’s search time is increased if the transmitter(s) can not be reliably detected, which is exacerbated in the case of greater densities of obstacles. The agent will therefore spend time revisiting previously visited patches to reach what it perceives to be likely location of a transmitter. Despite this, the agent is still able to search the environment at least as well as the lawnmower agent in an empty-field environment shown in Figure 1.

The next observation of interest is that the agent is able to find transmitters faster as the obstacle density rises from 20% to 25%. As signals are then unlikely to be detected until the agent is within close proximity to the transmitters, the agent is much more likely to be within the vicinity of the transmitter when it begins the discovery phase. The agent still gains the benefit of being able to pass through areas quickly and discern whether a transmitter is likely to be in the local area or not.

On the other hand, the random agent is not affected significantly by the number of obstacles. Any advantage from reduced path options is lost to getting “stuck” in maze-like structures.

## 8. Discussion

Our results show that our algorithm performs significantly faster than the lawnmower (“dense” path) behaviour in simple environments, that the algorithm relies on hearing signals to significantly speed-up search, and that obstacles do not significantly reduce agent search times. This speed increase can be attributed both in part to the algorithm itself outperforming the lawnmower search agent, and the “radio advantage” given by the ability to detect transmitted signals.

In Figure 1 and Figure 2 we show that despite varying transmitter properties, by making the assumption of a least favourable transmitter configuration the agent is able to outperform the random agent in every scenario tested and match or outperform the lawnmower search agent with a predefined search path. The advantage of IEB diminishes as the algorithm is not able to uniquely identify transmitters and the advantage of detecting targets is lost. In this case, we show that the algorithm is still able to outperform the lawnmower agent in every tested case. We note that when observing the IEB agent’s behaviour in a zero-transmitter case, it performs a behaviour similar to the lawnmower agent, but tends to prefer to spiral inwards instead. With greater densities of transmitters and with unfavourable transmitter signal configuration, we expect to see greater losses in performance of the IEB agent, perhaps even underperforming when compared to the lawnmower agent.

This property is useful in the context of SAR as it may not be known in advance what types of devices a victim may have or how many may be in the vicinity of one another. In reality, we should be able to give the agent a more reasonable expectation of expected transmitter properties based on local laws regarding transmit powers and the properties of expected transmitters a person is likely to carry, such as that of cellular, WiFi, or Bluetooth transmitters.

One of the advantages of the IEB algorithm is that it is able to navigate through various unknown densities of obstacles, something that is not greatly focused on in previous works. In Figure 3 we show that increasing the environment complexity by introducing obstacles does not dramatically increase search time, despite the obstacles occluding the transmitter signal. Increasing the environment complexity significantly even decreases time to search, as signals are more likely to be heard closer to the transmitter and when they are heard the agent is likely much closer to the transmitter origin, which in turn biases the agent’s search paths.

In the context of SAR, we must consider the environment has unknown complexity. For example, during an earthquake it is reasonable to expect the environment to dramatically change even if it was previously modelled [45]. The simple no-obstacle scenario where line of sight is possible is unlikely to be a scenario where a SAR team requires help searching. Instead, the deployment of such an agent would likely be over a vast space, where structures may have collapsed or the terrain varies greatly. Our results suggest that increasing complexity of the environment does not dramatically increase the agents search time, even helping it to search more efficiently in some extreme cases.

A disadvantage of the IEB algorithm is that although the average MTS is decreased when compared to another algorithm such as a lawnmower agent, we recognise that there are edge cases where the IEB agent may take longer to locate search victims in individual scenarios. As mentioned previously, we consider this risk somewhat mitigated by having a simpler agent search a “dense" path, which is also required in any case for locating search victims that do not carry some form of detectable wireless transmitter.

When compared to the work of Lanillos et al. [30], we showed that our IEB algorithm is able to choose paths that quickly discount large areas where a transmitter should not exist. We also showed that our algorithm is able to navigate and search complex environments and handle multiple transmitters of unknown properties.

## 9. Conclusions

In this paper, we propose an algorithm for searching large spaces with few a priori information. The agent searches for one or more objectives with minimal assumptions about the nature of the goal. The agent is able to simultaneously explore and search the environment, with the goal of maximising information revealed about the location of transmitters in the near future.

In future works, we would like to further explore the single agent case in the SAR context. One idea we are seek to explore is to allow the ability to uniquely detect transmitters, eliminating the need for the *discovery* phase in the IEB search algorithm and reducing the overall complexity. Equally, angle of attack (AoA) with some associated probability of angle would allow for more focused belief values. If we are able to decode a commonly used signal, it is likely we would be able to find a transmitted unique identifier. This would allow us to efficiently track multiple transmitters in an arbitrarily large environment.

Another research area of interesting is to look to propose better paths, without pruning potentially good paths from the agent’s search. One such method would be to explore UCT-tree search [39]. So far, we assumed the problem is 2D, but we would like to expand our work into 3D space to allow the searching of more complex environments, such as the internals of a building or to be able to make the decision to search the outside of a structure from different perspectives. To increase the search space to a 3D model, an improved pruning algorithm would be required to keep computation reasonable.

One key assumption was also that the potential victims involved do not move in the environment, which is not necessarily the case. In this case we would need decay our confidence that a transmitter is in a given location and increase the confidence that it may be located in surrounding patches [46].

Additionally, the IEB agent makes no use of signal strength information as other algorithms do. Although this information is known not to be reliable, it may still probabilistically speed up search if a transmitter can be said to be a distance away with some confidence. The ability to derive an approximate angle of the received signal would also greatly reduce the search space significantly.

In future experiments, the authors intend to decouple the “radio advantage" from the IEB agent’s fundamental search time without the ability to observe transmitters. We would also like to investigate overall cover time to visit every patch, an important measurement in the SAR problem space.

## Figures and Tables

**Table 1:** A list of fixed parameters used in experiments.

Symbol	Value	Description
*v*	0.5 m/s	The agent velocity
$T_{s}$	4 s	The time taken to move from one patch to another
*l*	2 m	The length of a given patch
*n*	10	The lookahead value of the IEB algorithm
paths	5000	The number of sampled paths for each iteration
max ticks	500,000	Maximum number of ticks for the IEB simulation (derived experimentally)
$L_{w}$	500	Environment width
$L_{h}$	500	Environment height
total patches	250,000	Total number of patches in the environment
